# Supplementary material for: Single trial neuronal activity dynamics of attentional intensity in monkey visual area V4
Source: Nat Commun. 2021 Mar 31;12:2003. doi: 10.1038/s41467-021-22281-2 (PMC8012644; doi:10.1038/s41467-021-22281-2)
Supplement: Supplementary file 1 — Supplementary Information [file 41467_2021_22281_MOESM1_ESM.pdf]

# SUPPLEMENTARY INFORMATION

## Supplementary Tables

**Supplementary Table 1. Modulation indices of attentional intensity.** Modulation indices of attentional intensity on behavioral and neural correlates, computed across intensity conditions.

| **Modulation Index** | **Mean ± SEM** | **p-value** |
|---|---|---|
| Reward (N = 24) | 0.598 ± 0.015 | p = $10^{-21}$ (two-sided t-test) |
| Pupil area (N = 24) | 0.102 ± 0.013 | p = $10^{-7}$ (two-sided t-test) |
| Behavioral d' (N = 24) | 0.265 ± 0.011 | p = $10^{-16}$ (two-sided t-test) |
| Microsaccades towards stimulus location (0-200 ms, N = 24) | –0.046 ± 0.068 | p = 0.53(two-sided t-test) |
| Neuronal d' (n = 970) | 0.130 ± 0.004 | p = $10^{-153}$ (two-sided t-test) |
| Fano factor | –0.009 ± 0.001 | p = $10^{-26}$ (two-sided t-test) |
| Spike-count correlation | –0.037 ± 0.003 | p = $10^{-30}$ (two-sided t-test) |

**Supplementary Table 2. Mean behavioral hit and false alarm (FA)**

| | Hit rate (%) (Mean ± SEM) | | False alarm rate (%) (Mean ± SEM) | |
|---|---|---|---|---|
| | Small reward | Large reward | Small reward | Large reward |
| Monkey P (N = 9) | 85.9 ± 2.2 | 92.8 ± 0.7 | 34.7 ± 2.4 | 13.1 ± 1.4 |
| Monkey S (N = 15) | 79.1 ± 1.4 | 89.1 ± 1.0 | 30.3 ± 1.4 | 15.4 ± 1.3 |

**Supplementary Table 3. Spatial RF location and size**

| | RF eccentricity (°) Mean ± SEM | RF polar angle (°) Mean ± SEM | RF size, sigma (°) Mean ± SEM |
|---|---|---|---|
| Monkey P (n = 66) | 2.8 ± 0.1 | 132.4 ± 2.6 | 1.13 ± 0.05 |
| Monkey S (n = 81) | 2.7 ± 0.1 | 108.8 ± 3.2 | 1.18 ± 0.06 |

**Supplementary Table 4. Modulation of behavioral, physiological and neurophysiological responses during early and late trials within a session.**

| | Reward size | Early trials | Late trials | ANOVA factors | | | |
|---|---|---|---|---|---|---|---|
| | **Monkey P** | | | | | | |
| | | | | Attention intensity | Trial timing | Intensity-by-time interaction | |
| Behavioral d’ | Small | 1.51 ± 0.13 | 1.59 ± 0.11 | F(1,32) = 100.94 (p = $10^{-4}$) | F(1,32) = 2.96 (p = 0.09) | F(1,32) = 0.7 (p = 0.4) | Two-way ANOVA |
| | Large | 2.45 ± 0.08 | 2.72 ± 0.08 | | | | |
| %Aborted trials | Small | 46.9 ± 3.5 | 56.3 ± 4.2 | F(1,32) = 36.9 (p = $10^{-4}$) | F(1,32) = 3.41 (p = 0.07) | F(1,32) = 1.43 (p = 0.24) | Two-way ANOVA |
| | Large | 31.9 ± 1.9 | 33.9 ± 1.9 | | | | |
| Pupil area | Small | 1.043 ± 0.007 | 0.947 ± 0.011 | F(1,159) = 108.86 (p = $10^{-4}$) | F(1,159) = 160.26 (p = $10^{-4}$) | F(1,159) = 1.77 (p = 0.18) | Two-way ANOVA |
| | Large | 1.143 ± 0.014 | 1.027 ± 0.008 | | | | |
| V4 spike rate | Small | 0.807 ± 0.008 | 0.764 ± 0.010 | F(1,16267) = 67.84 (p = $10^{-15}$) | F(1,16267) = 67.44 (p = $10^{-15}$) | F(1,16267) = 3.01 (p = 0.08) | Three-factor ANOVA |
| | Large | 0.927 ± 0.006 | 0.843 ± 0.009 | | | | |
| | **Monkey S** | | | | | | |
| | Reward size | Early trials | Late trials | ANOVA factors | | | |
| | | | | Attention intensity | Trial timing | Intensity-by-time interaction | |
| Behavioral d’ | Small | 1.47 ± 0.07 | 1.25 ± 0.07 | F(1,56) = 162.6 (p = $10^{-4}$) | F(1,56) = 1.38 (p = 0.24) | F(1,56) = 3.55 (p = 0.06) | Two-way ANOVA |
| | Large | 2.26 ± 0.07 | 2.31 ± 0.07 | | | | |
| %Aborted trials | Small | 54.4 ± 2.2 | 58.5 ± 1.8 | F(1,56) = 7.56 (p = 0.008) | F(1,56) = 0.05 (p = 0.83) | F(1,56) = 4.12 (p = 0.05) | Two-way ANOVA |
| | Large | 53.2 ± 1.7 | 49.9 ± 1.3 | | | | |
| Pupil area | Small | 1.004 ± 0.005 | 0.956 ± 0.005 | F(1,250) = 32.85 (p = $10^{-4}$) | F(1,250) = 96.4 (p = $10^{-4}$) | F(1,250) = 0.11 (p = 0.74) | Two-way ANOVA |
| | Large | 1.038 ± 0.006 | 0.984 ± 0.005 | | | | |
| V4 spike rate | Small | 0.851 ± 0.007 | 0.825 ± 0.006 | F(1,22516) = 16.08 (p = $10^{-4}$) | F(1,22516) = 21.34 (p = $10^{-4}$) | F(1,22516) = 0.5 (p = 0.48) | Three-factor ANOVA |
| | Large | 0.892 ± 0.007 | 0.886 ± 0.006 | | | | |

**Supplementary Table 5. Single-trial decay/rise constant (*τ*) for behavior sensitivity (*d'*), physiology and V4 neurophysiology in response to reward changes.**

| | Behavior ($\tau_{d'}$, 95% CI) | | Pupil area ($\tau_{pupil}$, 95% CI) | | V4 spiking ($\tau_{neuron}$, 95% CI) | |
|---|---|---|---|---|---|---|
| | small→large | large→small | small→large | large→small | small→large | large→small |
| Monkey P ($N_{small}$ = 77; $N_{Large}$ = 86) | 11.4 (5.6, 17.3) | 1.1 (–0.1, 2.3) | 2.7 (2.2, 3.1) | 13.7 (12.4, 15.0) | 2.6 (1.5, 3.7) | 11.1 (8.4, 13.8) |
| Monkey S ($N_{small}$ = 128; $N_{Large}$ = 126) | 15.6 (8.5, 22.6) | 0.8 (–0.1, 1.7) | 5.1 (4.1, 6.0) | 8.7 (6.9, 10.5) | 1.4 (–0.4, 3.1) | 6.3 (3.4, 9.1) |

## Supplementary Figures

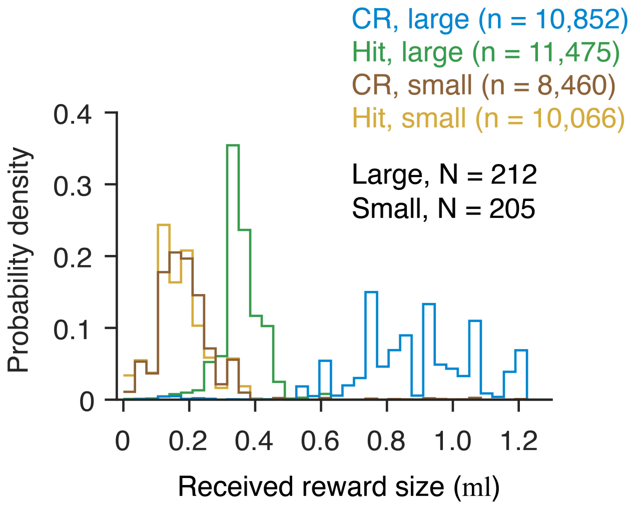


**Supplementary Figure 1. Distribution of received rewards.** Distributions of trial-by-trial received rewards (normalized within each session) for correct responses, hits (Hit) and correct rejections (CR) across small (N = 205) and large (N = 212) reward blocks.

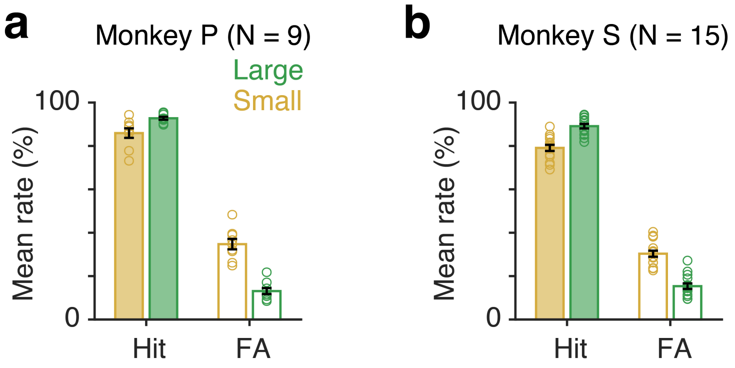


**Supplementary Figure 2. Mean behavioral performance across sessions.** Hit and FA rates during small and large reward blocks for monkey P (N = 9 sessions). **b** Same as in **a** for monkey S (N = 15 sessions). Circles, individual sessions. Error bars, mean ± SEM.

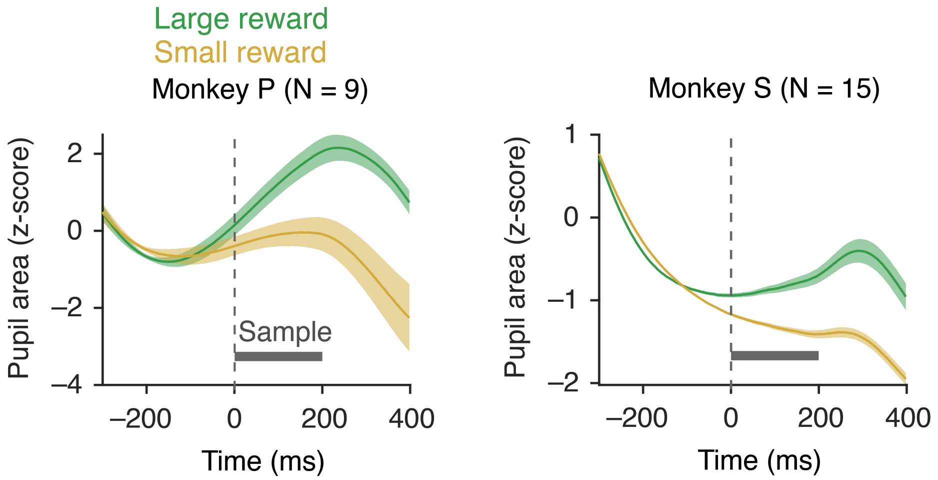


**Supplementary Figure 3. Session averaged stimulus-evoked pupil area for small and large rewards.** Pupil areas on single trials were first aligned with the sample stimulus onset. Time course of mean pupil area within a session was z-scored with respect to the pre-sample fixation period (–400 to 0 ms) separately for two different reward sizes. Left, monkey P (N = 9). Right, monkey S (N = 15). Error bars, mean ± SEM.

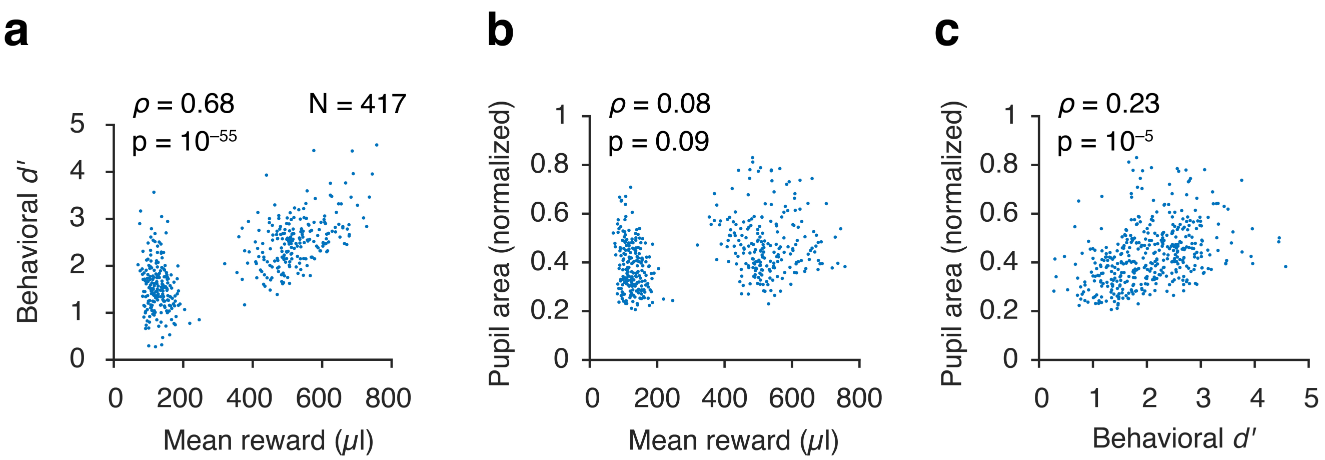


**Supplementary Figure 4. Correlations between block-by-block trial averaged behavioral *d'*, pupil area and reward size.** (**a-c**) Distributions of mean reward size versus behavioral *d'* (a); reward size versus pupil area (b) and behavioral *d'* versus pupil area (c) across all reward-blocks (N = 417; Spearman partial correlation with two-sided t-test).

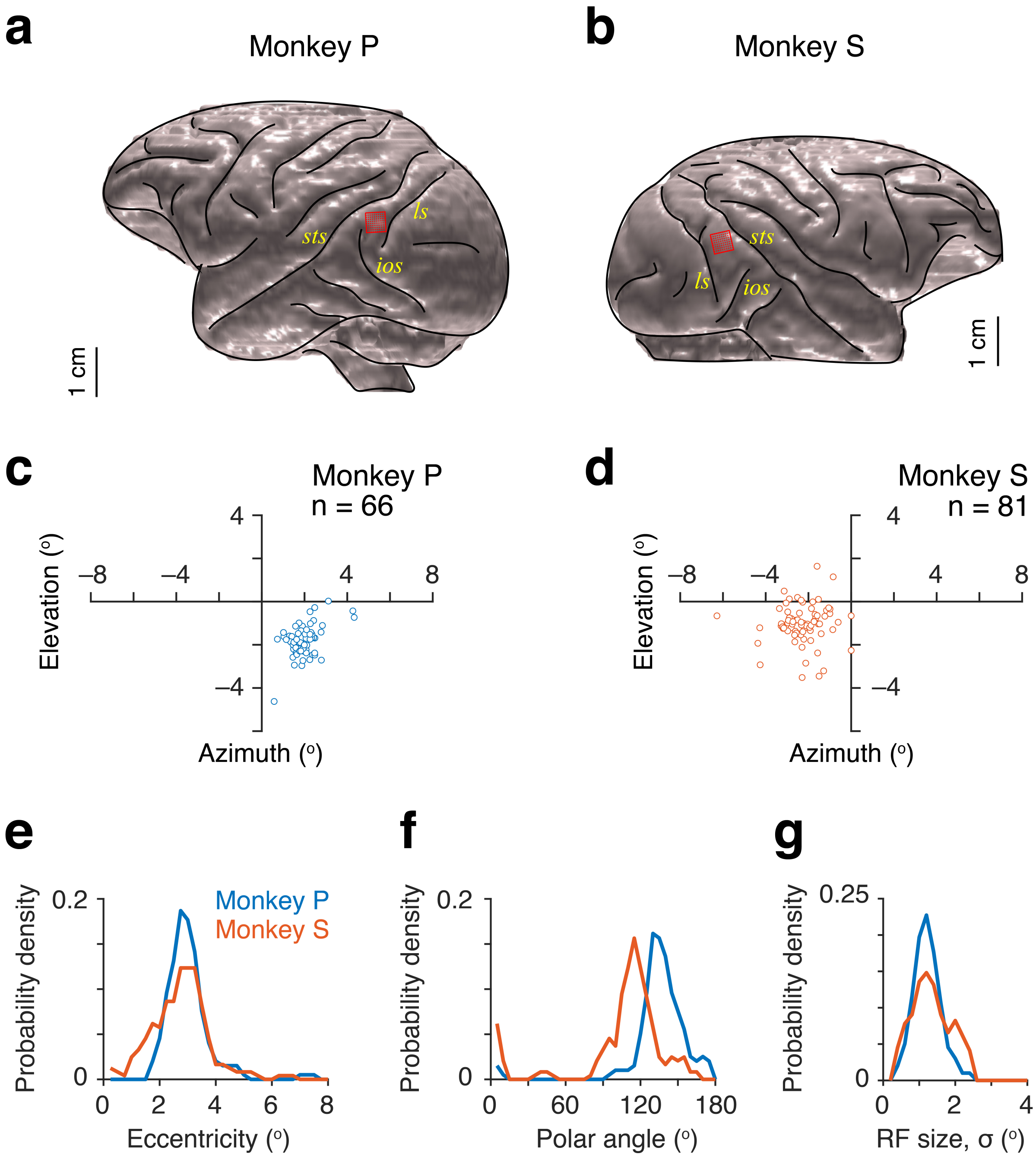


**Supplementary Figure 5. Electrode array placements. a-b** Reconstructed brain structural MRI of monkey P (a) and monkey S (b). *sts*, Superior temporal sulcus; *ls*, lunate sulcus; *ios*, inferior occipital sulcus. Red box with grids, 10x10 electrode array. **c-d** Spatial receptive field (RF) centers of all unique units (only one unit from each electrode contact) from monkey P (c) and monkey S (d). **e-g** Distributions of RF eccentricities (e), polar angles (f) and RF size (standard deviation, sigma; g) for both monkeys. There is no difference between the eccentricities ($p = 0.18$, two-sided ranksum test; e) and RF sizes ($p = 0.76$, two-sided ranksum test; g) of recorded units in the two monkeys. RF Polar angles between the two animals differed significantly ($p = 10^{-11}$, two-sided ranksum test; f).

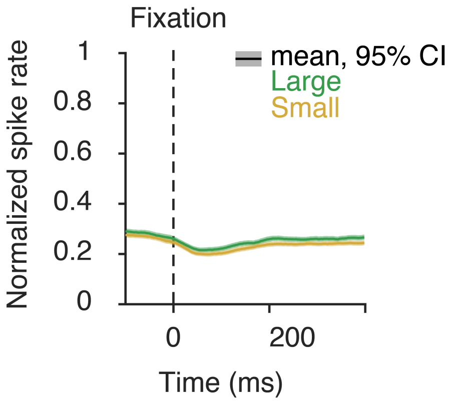


**Supplementary Figure 6. Population peri-stimulus time histogram of spike rates of V4 units aligned to fixation.** Spike rates of each neuron (n = 970) were normalized to its peak response within 60 - 260 ms from sample stimulus onset similar to Figure 3b-c. Error bars, 95% confidence intervals (bootstrap, n = $10^4$). Mean spike counts over 200 ms (0 - 200 ms from fixation) did not differ across population of V4 units [mean ± sem, for all units, 5.16 ± 1.5 $s^{-1}$ (small reward), 5.51 ± 0.15 $s^{-1}$ (large reward), p = 0.06; for single units, 4.63 ± 0.27 $s^{-1}$ (small reward), 4.79 ± 0.28 $s^{-1}$ (large reward), p = 0.72; for multiunits, 5.40 ± 0.17 $s^{-1}$ (small reward), 5.82 ± 0.18 $s^{-1}$ (large reward), p = 0.04; two-sided ranksum test].

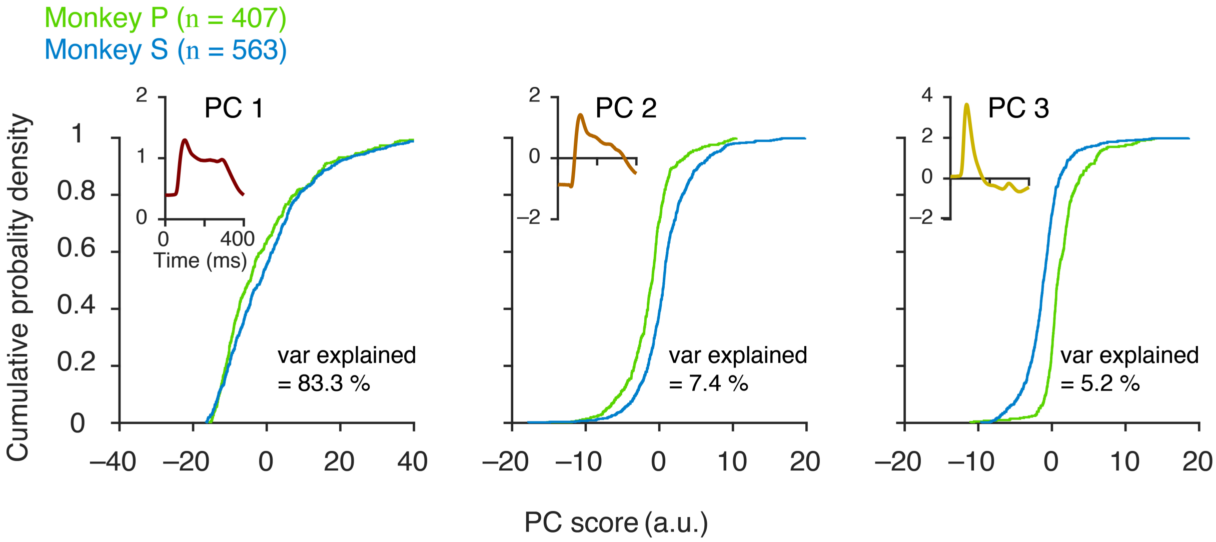


**Supplementary Figure 7. Comparison of principal components of spike count PSTHs between animals.** Principal components analysis on spike peri-stimulus time histograms of all recorded neurons' (PSTH; 0 - 400 ms from sample stimulus onset during all block of trials; n = 970). *Insets*, First three PCs. Bottom, Cumulative probability densities of PC scores (PC1, PC2 and PC3) for neurons in monkey P (green, n = 407) and monkey S (blue, n = 563). PC1 and PC2 primarily capture sustained spike response whereas PC3 associates with transient peak response. Monkey S has higher PC scores for PC1 and PC2 compared to monkey P (PC1 score, p = 0.006; PC2 score, p = $10^{-23}$; two-sided Kruskal-Wallis test). In contrast, monkey P has higher PC3 scores compared to monkey S (PC2 score, p = $10^{-52}$; two-sided Kruskal-Wallis test).

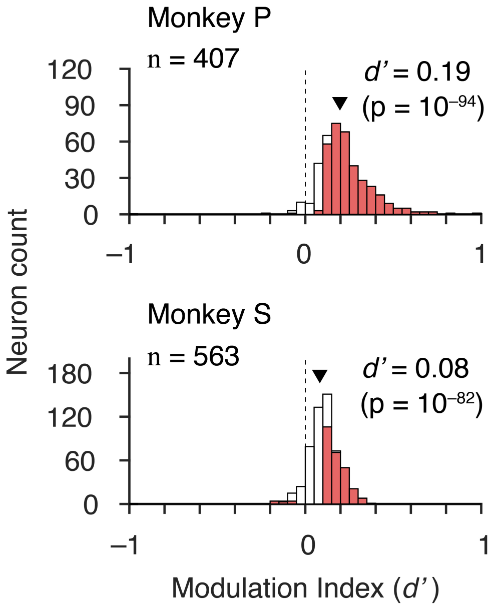


**Supplementary Figure 8. Distribution of neuronal modulation indices (*d'*).** Distribution of neuronal *d'* for monkey P (top) and monkey S (bottom). Red bars, neurons with *d'* values significantly different from zero (Monkey P, $n_{d' > 0} = 338/407$, $n_{d' < 0} = 2/407$; Monkey S, $n_{d' > 0} = 257/563$, $n_{d' < 0} = 11/563$, $p < 0.05$; two-sided t-test). White bars, non-significant *d'*. Solid triangle, population *d'* across all units in a monkey (Monkey P, n = 407, $p = 10^{-94}$; Monkey S, n = 563, $p = 10^{-82}$, two-sided t-test).

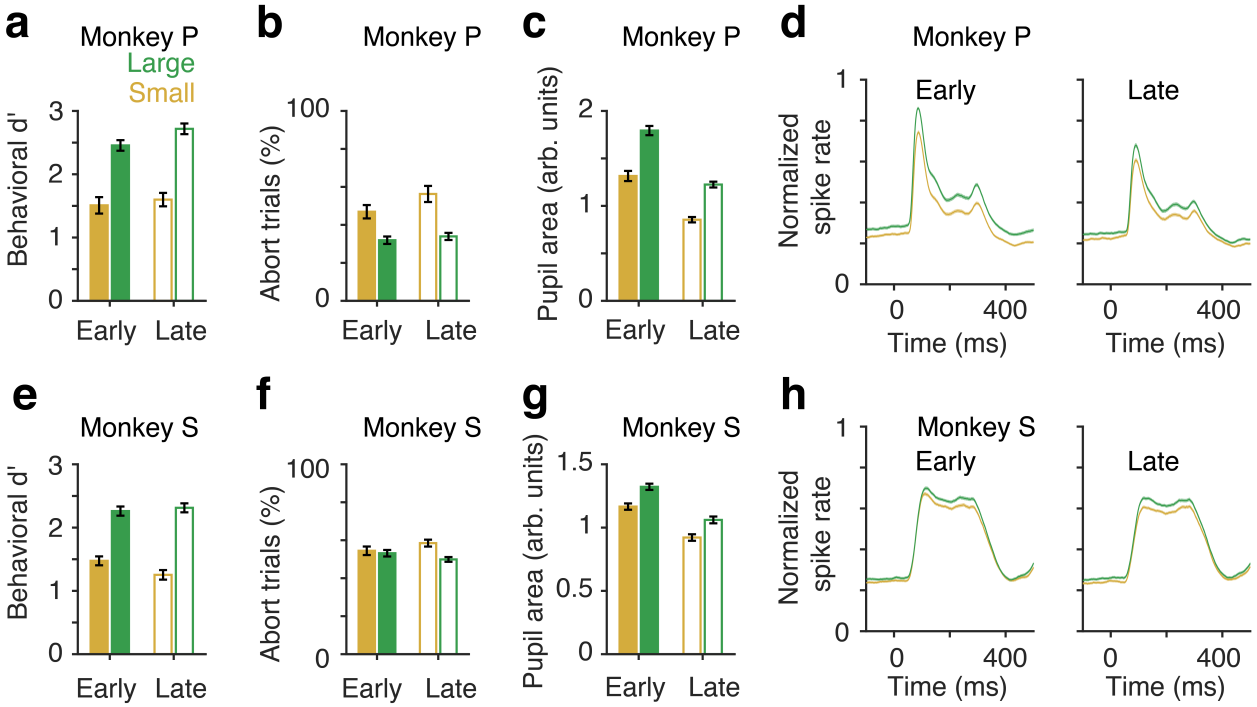


**Supplementary Figure 9. Comparing behavioral (*d'*), physiological (pupil area) and neuronal modulations between early and late halves of trials within a session.** Trials were divided into two halves, early trials and late trials in every session for each monkey. **a, e** Behavioral sensitivity (*d'*) across blocks during early and late trials with reward changes (#sessions, N = 9 for monkey P; N = 15 for monkey S). Except the reward size (small versus large), there was no significant effect of within session trial time (early versus late) and reward-by-trial time interaction on behavioral *d'* (monkey P, reward size, $p = 10^{-4}$, $F_{(1, 32)} = 100.9$; trial time, $p = 0.09$, $F_{(1, 32)} = 2.96$; reward-by-trial time interaction, $p = 0.4$, $F_{(1, 32)} = 0.7$; monkey S, reward size, $p = 10^{-4}$, $F_{(1, 56)} = 162.6$; trial time, $p = 0.24$, $F_{(1, 56)} = 1.4$; reward-by-trial time interaction, $p = 0.06$, $F_{(1, 56)} = 3.5$; two way ANOVA). **b, f** Mean rate of aborted trials (%) across blocks during early and late trials with rewards changes. Other than the reward size, there was no significant effect of within session trial time and reward-by-trial time interaction on the rate of aborted trial (monkey P, reward size, $p = 10^{-4}$, $F_{(1, 32)} = 36.9$; trial time, $p = 0.07$, $F_{(1, 32)} = 3.4$; reward-by-trial time interaction, $p = 0.24$, $F_{(1, 32)} = 1.4$; monkey S, reward size, $p = 0.008$, $F_{(1, 56)} = 7.6$; trial time, $p = 0.8$, $F_{(1, 56)} = 0.05$; reward-by-trial time interaction, $p = 0.05$, $F_{(1, 56)} = 4.1$; two way ANOVA). **c, g** Mean pupil area during the sample stimulus period ((#blocks, $N_{small} = 77$, $N_{large} = 86$, monkey P; $N_{small} = 128$, $N_{large} = 126$, monkey S). There was no significant effect of reward-by-trial time interaction on pupil areas (monkey P, reward size, $p = 10^{-4}$, $F_{(1, 159)} = 108.9$; trial time, $p = 10^{-4}$, $F_{(1, 159)} = 160.3$; reward-by-trial time interaction, $p = 0.18$, $F_{(1, 159)} = 1.8$; monkey S, reward size, $p = 10^{-4}$, $F_{(1, 250)} = 32.8$; trial time, $p = 10^{-4}$, $F_{(1, 250)} = 96.4$; reward-by-trial time interaction, $p = 0.73$, $F_{(1, 250)} = 0.1$; two way ANOVA). **d, h** PSTHs of V4 neuronal spike rates (#units, n = 407, monkey P; n = 563, monkey S; 20 ms bin). There was no significant effect of reward-by-trial time interaction on spike-counts (monkey P, reward size, $p = 10^{-15}$, $F_{(1, 16267)} = 67.8$; trial time, $p = 10^{-15}$, $F_{(1, 16267)} = 67.4$; reward-by-trial time interaction, $p = 0.08$, $F_{(1, 16267)} = 3.01$; monkey S, reward size, $p = 10^{-4}$, $F_{(1, 22516)} = 16.1$; trial time, $p = 10^{-5}$, $F_{(1, 22516)} = 21.3$; reward-by-trial time interaction, $p = 0.48$, $F_{(1, 22516)} = 0.5$; 3-factor way ANOVA). Error bars, mean ± SEM.

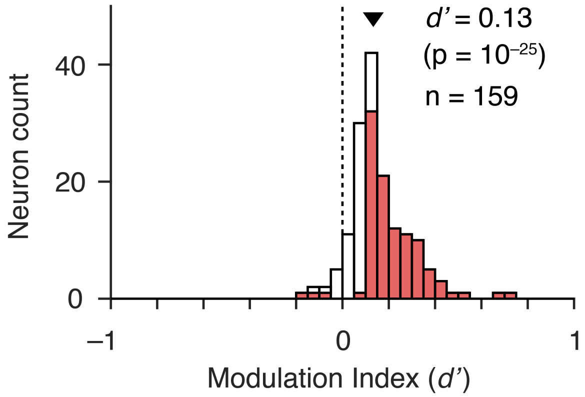


**Supplementary Figure 10. Distribution of neuronal modulation of unique units across recording sessions.** A unique unit was randomly selected from every electrode out of a multielectrode array (96 channel) across session. Distribution of neuronal *d'* of these unique units (n = 159) are plotted. Red bars, neurons with *d'* values significantly different from zero ($n_{d'>0}$ = 99/ 159, $n_{d'<0}$ = 3/ 159, $p < 0.05$; two-sided t-test). White bars, non-significant MI. Solid triangle, mean population MI across all unique units (n = 159, $p = 10^{-25}$; two-sided t-test).

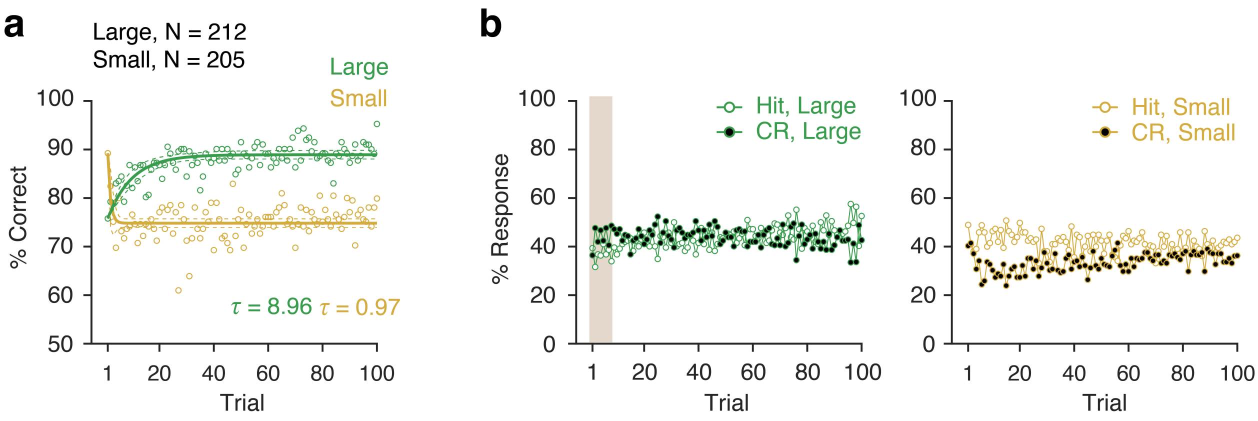


**Supplementary Figure 11. Trial-by trial behavioral performances, percent corrects.** Block averaged trial-by-trial percentage of correct responses (**a**), hits (*left*, b) and CRs (*right*, b) for large (N = 212) and small (N = 205) reward conditions. Color bar in (**b**) highlights 1st 10 trials after the reward switches from small to large. The slow rise of percent corrects on transition from small to large reward was associated with a slow increase in hits and a transient increase in CR (b**,** Supplementary Figure 12a) which led to a small increase in criterion and a slower rise of *d'* (Supplementary Figure 12b and 12c). The proportion of CRs within the first 10 trials was slightly higher compared to hits for small-to-large reward switch (CR = 45.5%, hit = 37.1%, p = 0.0003, two-sided t-test). Thus, a faster rise of rewards (Figure 5a) relative to percent correct and *d'* is primarily determined by reward size for CRs which is >2.5 fold larger than rewards size for hits (Supplementary Figure 1).

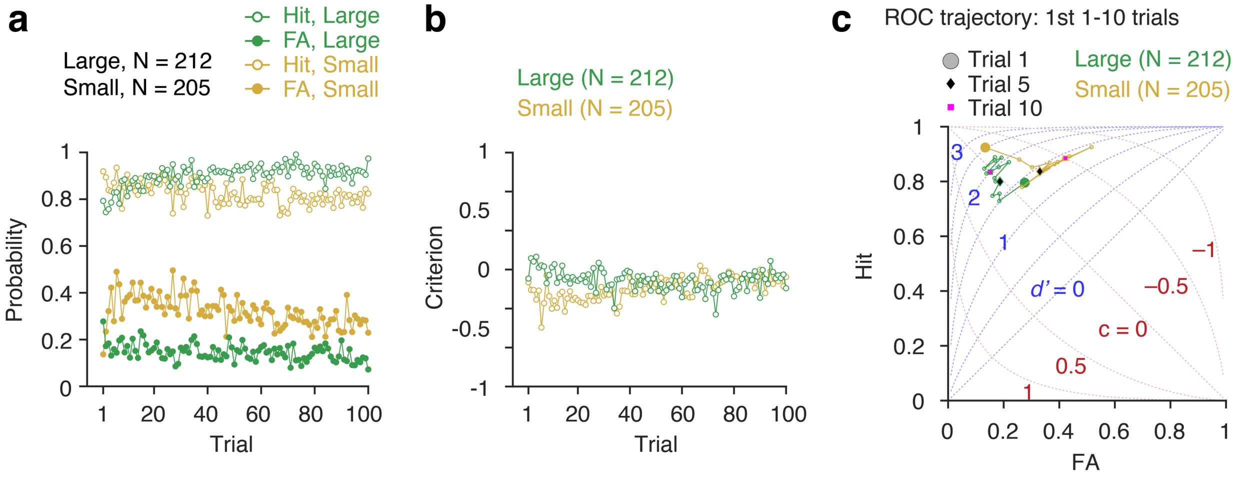


**Supplementary Figure 12. Trial-by-trial behavioral performances, response probabilities and criterion. a** Block averaged trial-by-trial probabilities of hit, miss, correct rejection and false alarm. **b** Block averaged trial-by-trial criterion for large (N = 212) and small (N = 205) reward conditions. **c** Trajectories of receiver operating characteristic (ROC) at the block transition. Filled circle, start of a block (trial 1). Diamond, fifth trial after the block transition. Square, tenth trial after the block transition.

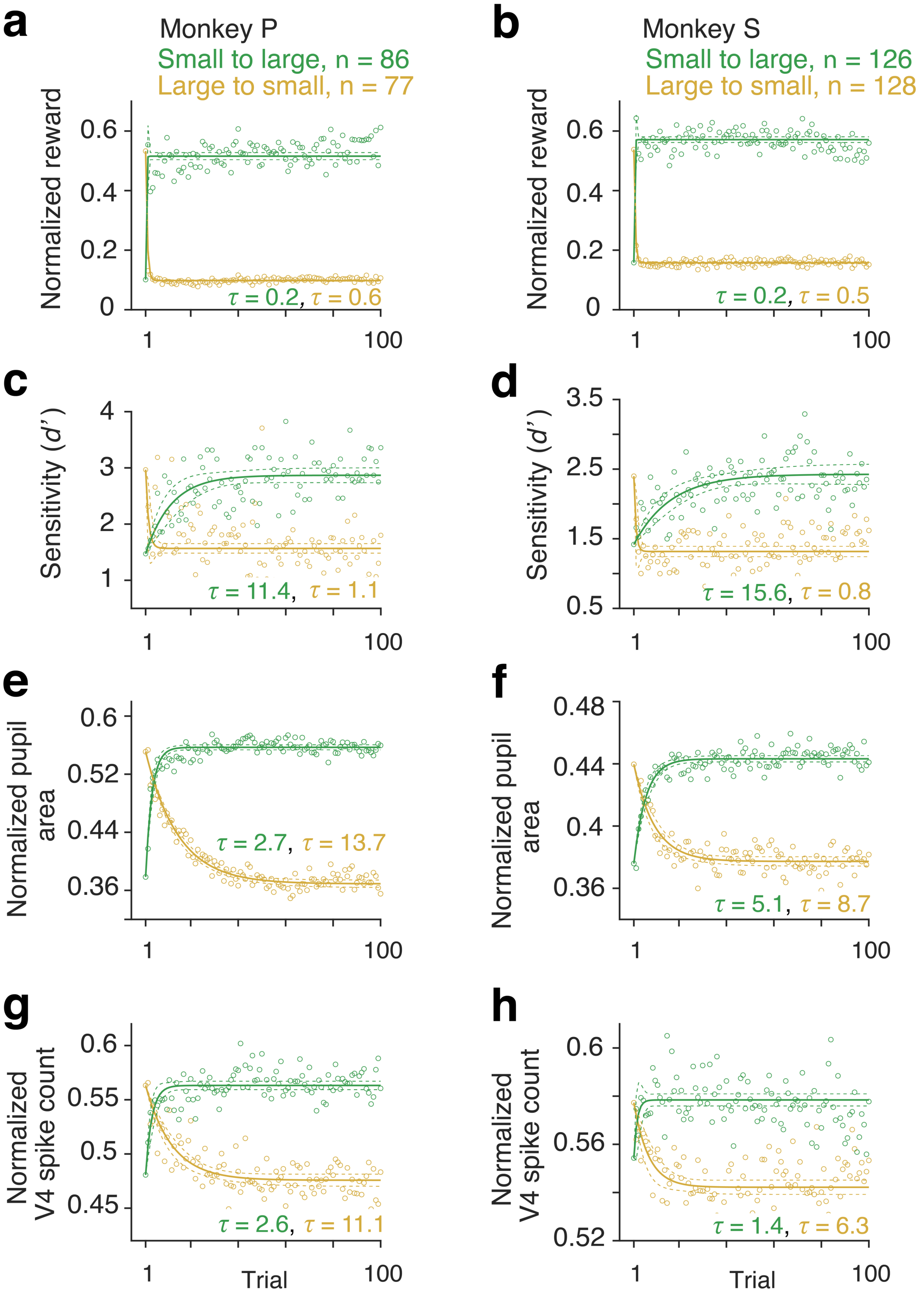


**Supplementary Figure 13.** Block averaged trial-by-trial dynamics of behavioral sensitivity (*d'*), pupil area and V4 neuronal spiking with reward changes for monkey P (**a**, **c**, **e** and **g**; #blocks, $N_{small}$ = 77, $N_{large}$ = 86) and monkey S (**b**, **d**, **f** and **h**; #blocks, $N_{small}$ = 128, $N_{large}$ = 126). Circles, observed data. Lines, single exponential fits. $\tau$, decay or rise constants. Trials are aligned with respect to the first correct trial following a block transition. Dashed lines, 95% confidence intervals around mean. **a, b** Received rewards. **c, d** Behavioral sensitivity (*d'*). **e, f** Normalized mean pupil area during sample stimulus period. **g, h** Normalized V4 spike counts across all recorded neurons (monkey P, n = 407; monkey S, n = 563).

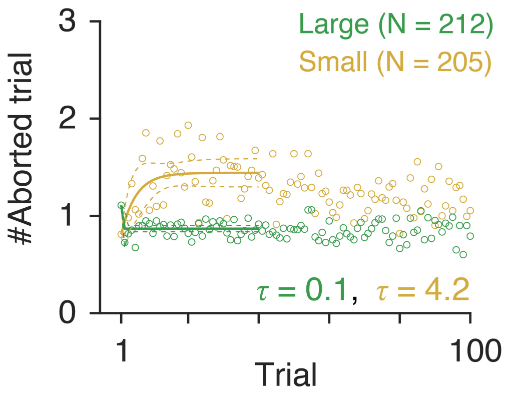


**Supplementary Figure 14. Trial-by-trial aborted trials.** Block averaged trial-by-trial aborted trials (fixation breaks) for large (N = 212) and small (N = 205) reward conditions. Circles, observed data. Lines, single exponential fits. $\tau$, decay or rise constants. Trials are aligned with respect to the first correct trial following block transition. Dashed lines, 95% confidence intervals around mean.

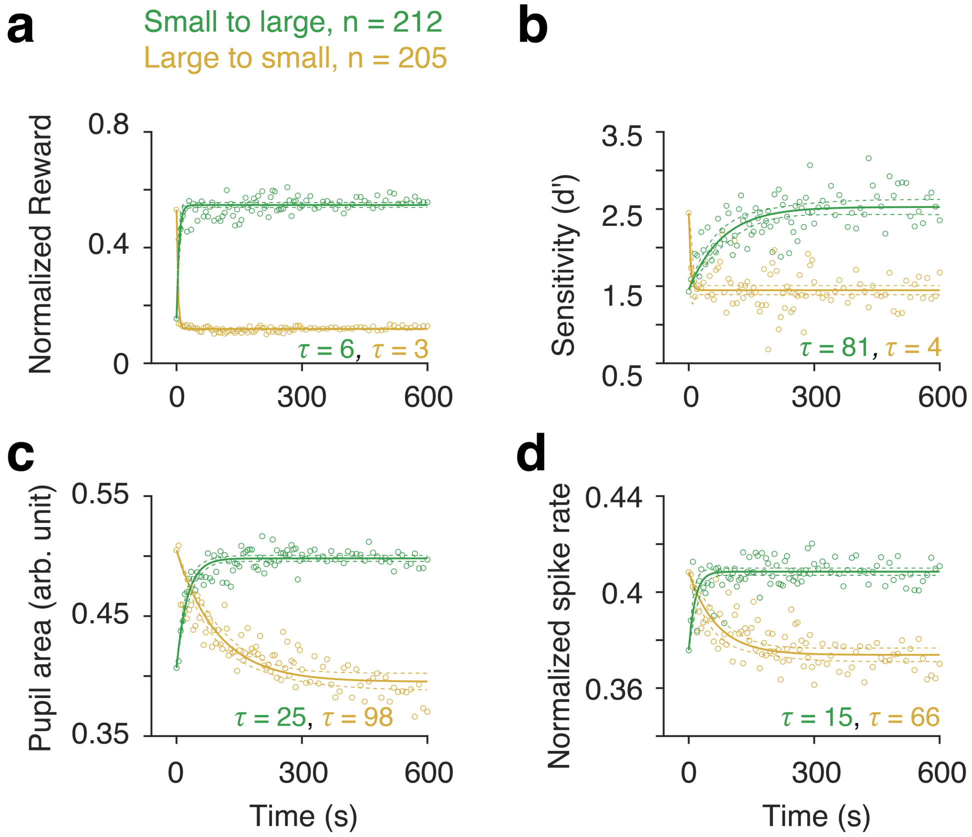


**Supplementary Figure 15.** Block averaged temporal dynamics of behavioral sensitivity (*d'*), pupil area and V4 neuronal spiking with reward changes as a function of time (#blocks, small, 205; large, 212; two monkeys). **a** Rewards received. **b** Behavioral sensitivity (*d'*). **c** Mean pupil area during sample stimulus. **d** Mean normalized spike counts across blocks and neurons (n = 970). $\tau$, decay or rise constants in seconds. Trials are aligned with respect to the first correct trial following block transition. Dashed lines, 95% confidence intervals around mean.

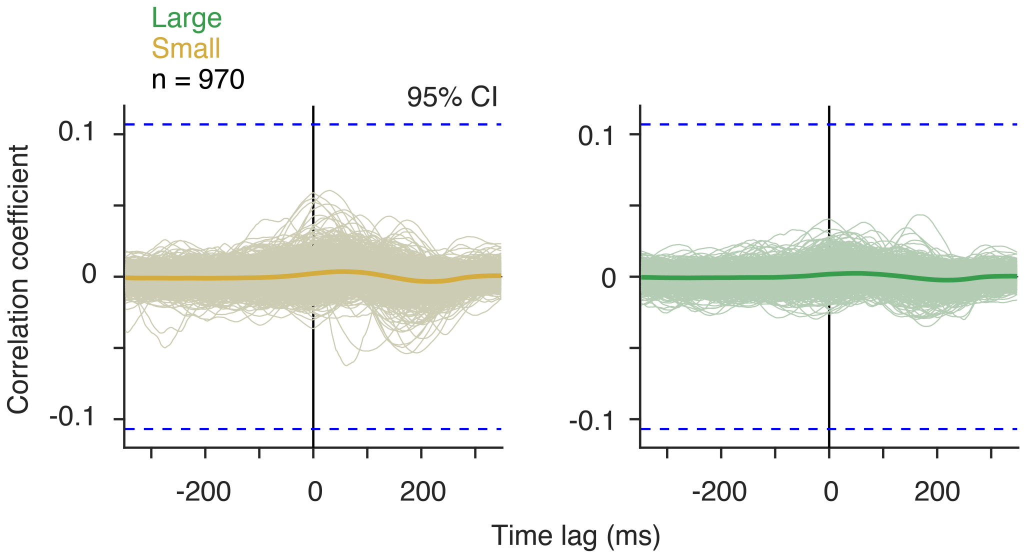


**Supplementary Figure 16. Cross-correlation between V4 spike rate and pupil area**. *Left*, Cross-correlations (subtracted from trial shuffled values) between pupil area and single trial V4 spike rates of individual V4 neurons (*thin lines*) and averaged across neurons (*thick lines*, n = 970) for small reward trials (–350 to 350 ms from sample stimulus on). Dotted lines, 95% confidence interval. A positive peak at a negative lag would have indicated that changes in V4 spiking followed changes in pupil area with same sign. Cross-correlations for large reward trials similar to the *Left*.

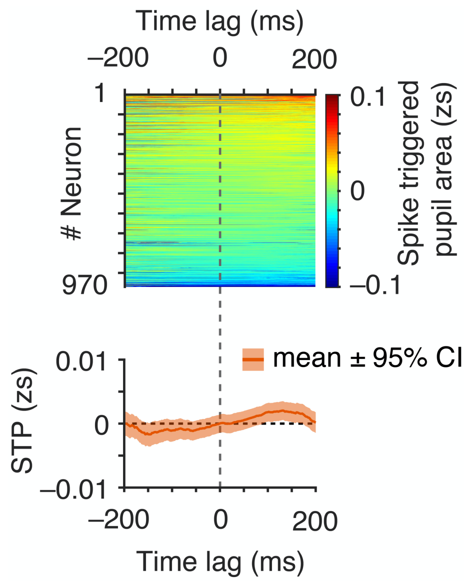


**Supplementary Figure 17. Spike triggered (STA) averaged pupil area**. STA-pupil area (subtracted from trial shuffled values) of individual V4 neurons (*top*) and averaged across neurons (*bottom*). Spikes within 400 ms period from sample stimulus onset (0 - 400 ms) were considered. Error bars, 95% confidence intervals (bootstrap, n = $10^4$).

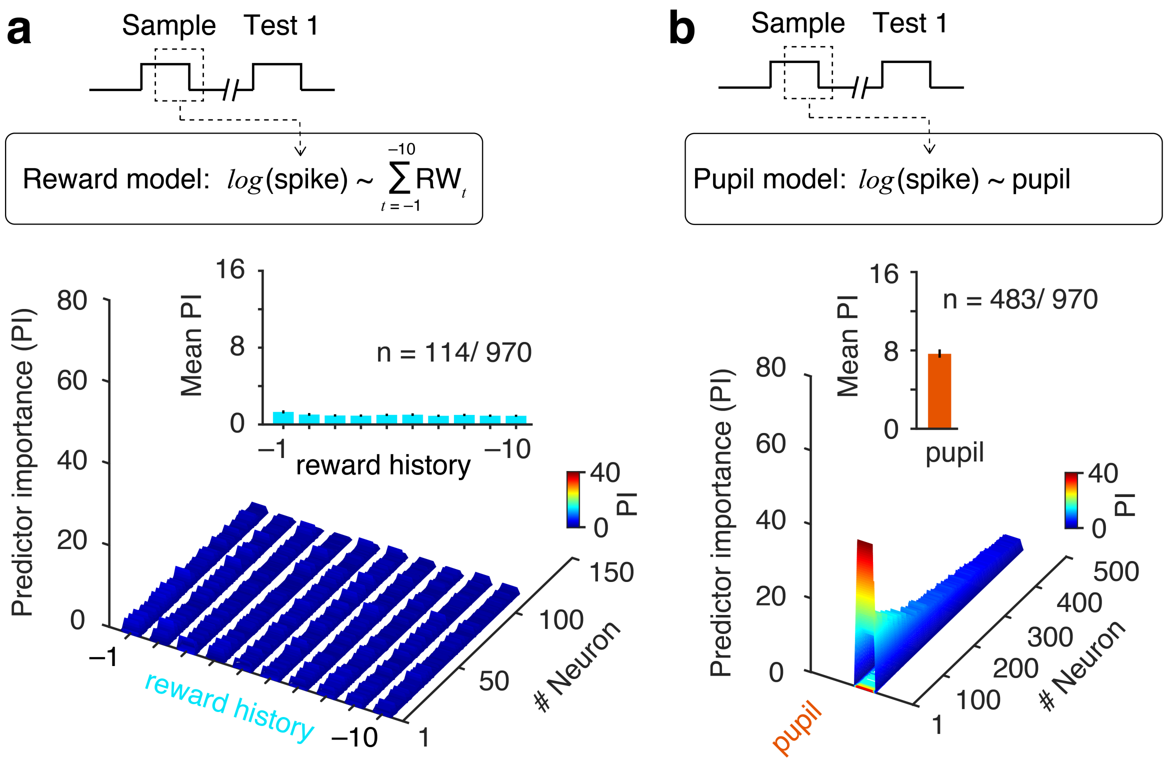


**Supplementary Figure 18. Comparison of different reduced GLM fits in Figure 6**. *Top* (**a, b**), Schematics of the analysis time window for spike counts (60 -260 ms from sample stimulus onset) used for GLM fits. **a** Reward model, based on reward history (10 immediate past received rewards). Data in the bar plot are presented as mean with 95% confidence intervals (bootstrap, n = $10^4$). **b** Pupil model, mean pupil area over 400 ms from stimulus onset. The same set of trials as in Figure 6 was used. *Bottom,* (a, b), predictor importance (PIs) that measures contributions of different predictor variables estimated by absolute standardized predictor coefficient values for all the neurons that were significantly fitted ($p < 0.05$; two-sided F-test). *Inset*, Mean predictor importance averaged across neurons. Data are presented as mean with 95% confidence intervals (bootstrap, n = $10^4$).

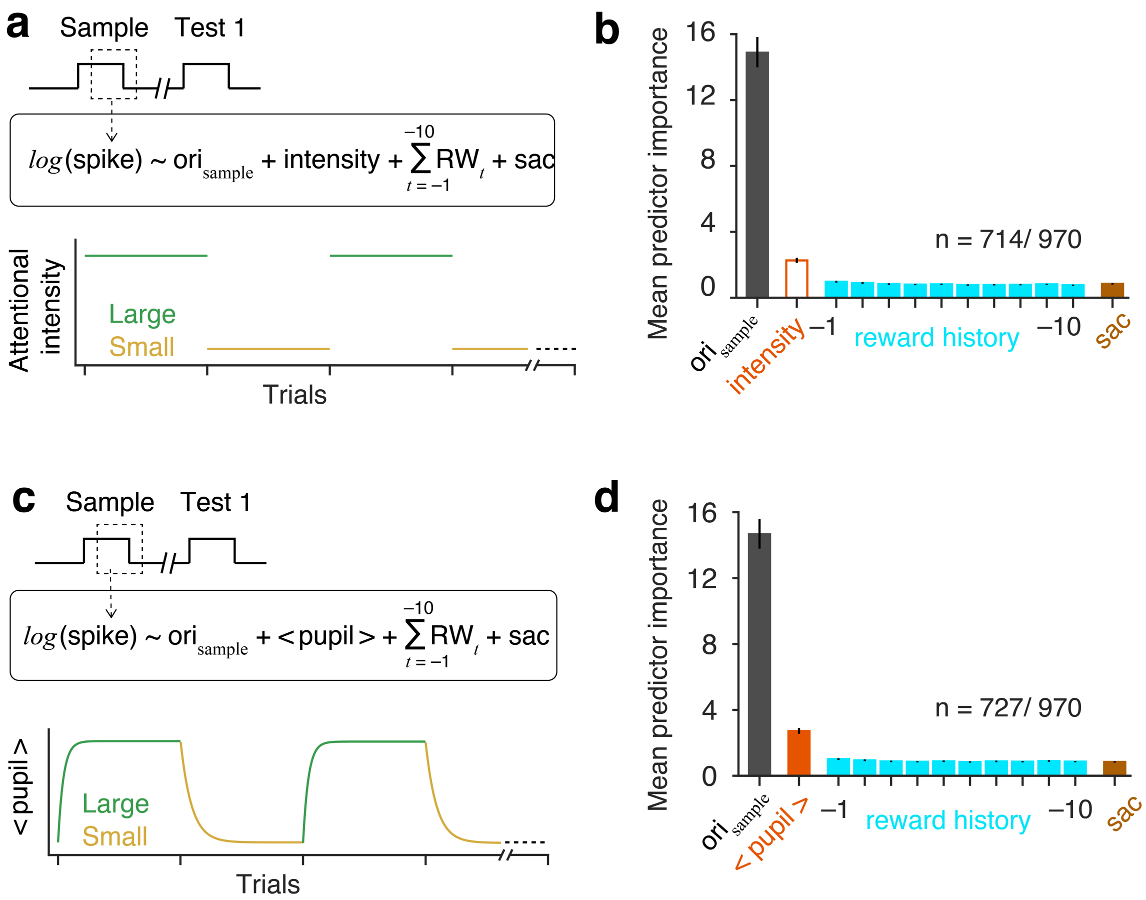


**Supplementary Figure 19. Alternate GLM fitting of spike counts during sample stimulus. a, b** Constant attentional intensity GLM: Spike counts (60 - 260 ms from sample onset) were fit an alternate complete GLM which contained stimulus Gabor orientation of sample stimulus, constant attentional intensity (categorical variable, either 'high' or 'low'; *bottom*, (a)), reward history (past 10 trials) and saccade response. Bar plot in (b) shows averaged predictor importance across neurons (n = 714). Data presented as mean with 95% confidence intervals (bootstrap, $n = 10^4$). **c** Averaged pupil area GLM: It contained stimulus Gabor orientation of sample stimulus, within session block averaged pupil area (*bottom*, (c)), reward history (past 10 trials) and saccade response. Pupil areas were averaged across large and small reward blocks separately to estimate within session single-trial-dynamics of pupil area. Thus, the variable < pupil > has average dynamics of block transitions, but lacks single trial values. **d** Averaged predictor importance for in (c). Data presented as mean with 95% confidence intervals (bootstrap, $n = 10^4$).

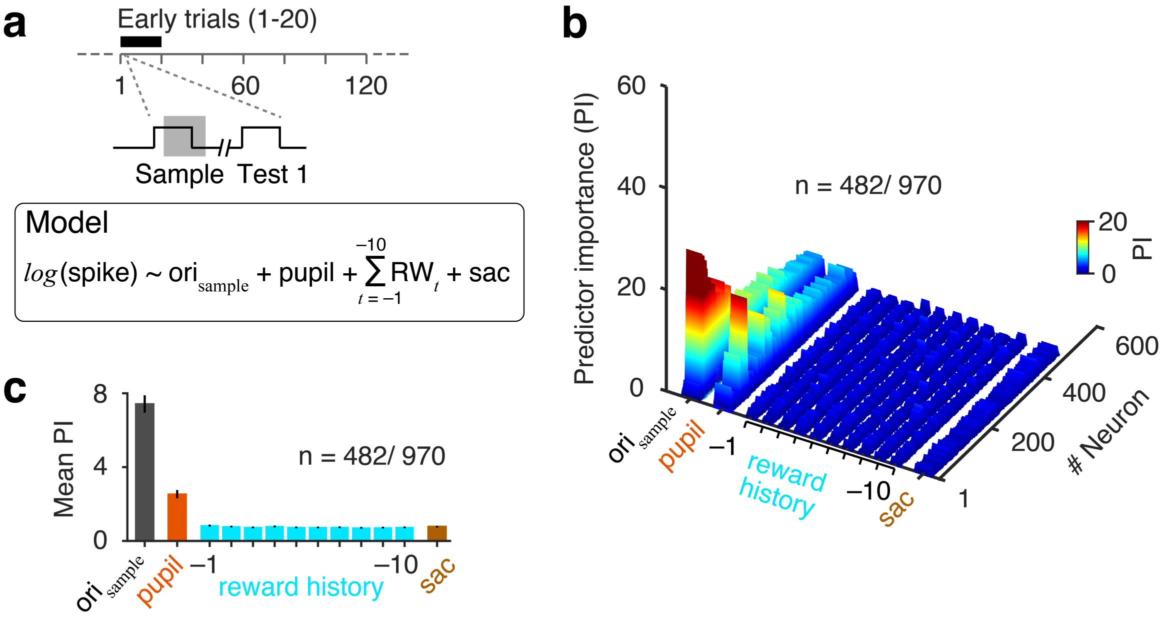


**Supplementary Figure 20. GLM fitting of spike counts during sample stimulus for the first 20 trials across all blocks. a** Complete GLM same as in Figure 6. Spike counts are over 60-260 ms from sample stimulus onset for first 20 trials from first correct response. **b** Colormap represents predictor importance (PIs) for every neuron fitted with the complete model ($p < 0.05$, n = 482/970; two-sided F test). PI measure contributions of different predictor variables estimated by absolute standardized predictor coefficient values. Neurons were sorted based on the R-squared values (Methods). **c** Averaged predictor importance across neurons (n = 482). Data presented as mean with 95% confidence intervals (bootstrap, $n = 10^4$).

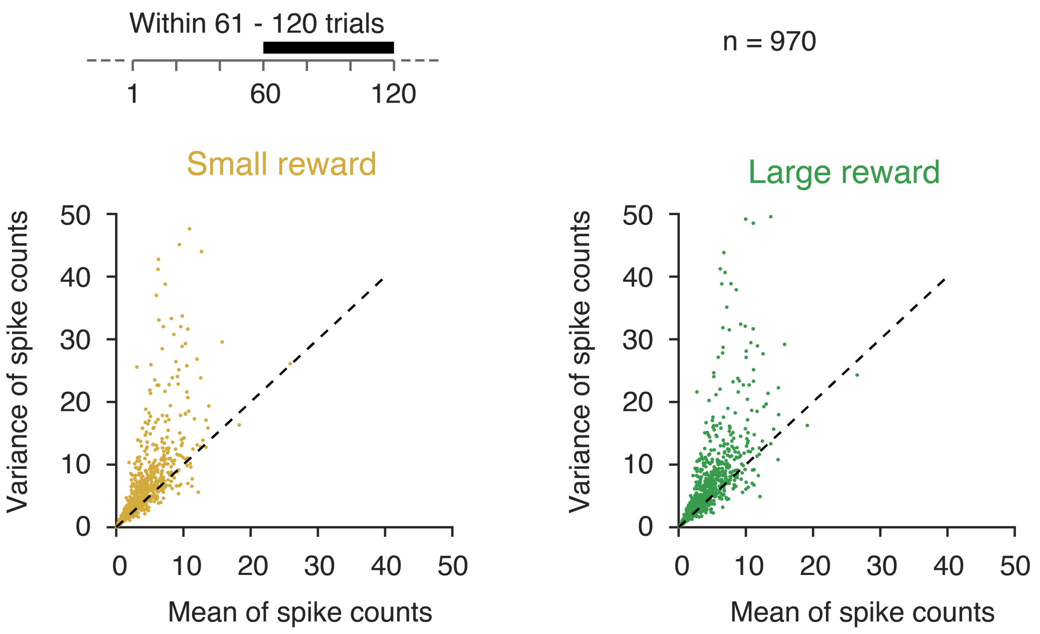


**Supplementary Figure 21. Variances and means of spike counts**. Each marker represents the mean and variance of spike counts over a period of 200 ms (60 to 260 ms from stimulus onset) of a neuron for the same sample stimulus orientation and steady state reward/attentional intensity (last 60 trials in every block). Dashed lines correspond to the expected mean and variance for a Poisson count process.

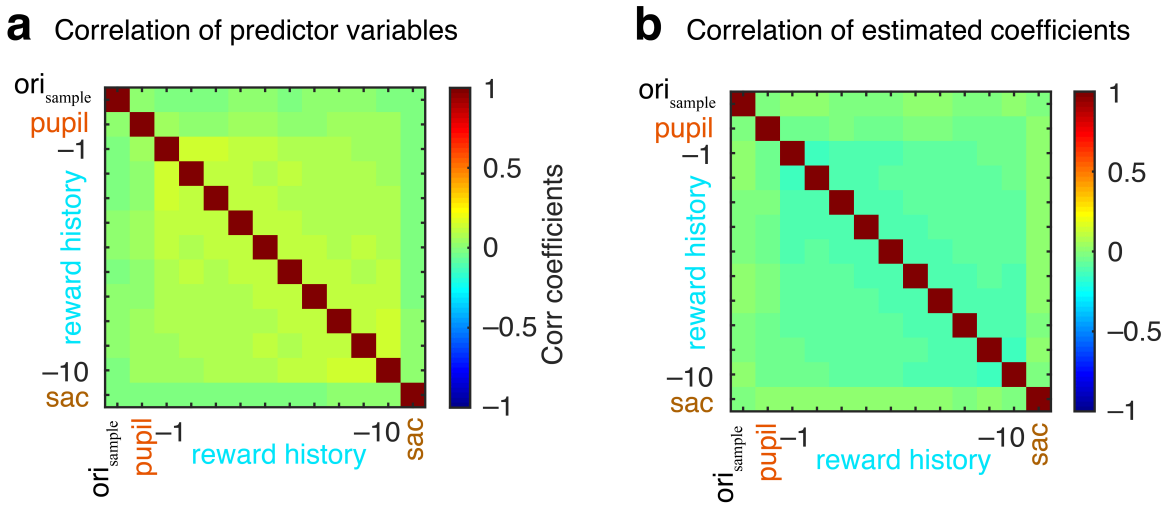


**Supplementary Figure 22. Single trial partial correlation matrices between all predictor variables during sample stimulus period (a) and between GLM fitted predictor coefficients (b).** Dataset includes all trials in all sessions (N = 24, two monkeys, **Figure 6**). There were no significant correlations among predictor variables or fitted coefficients ($p < 0.05$; partial correlation with two-sided t-test).

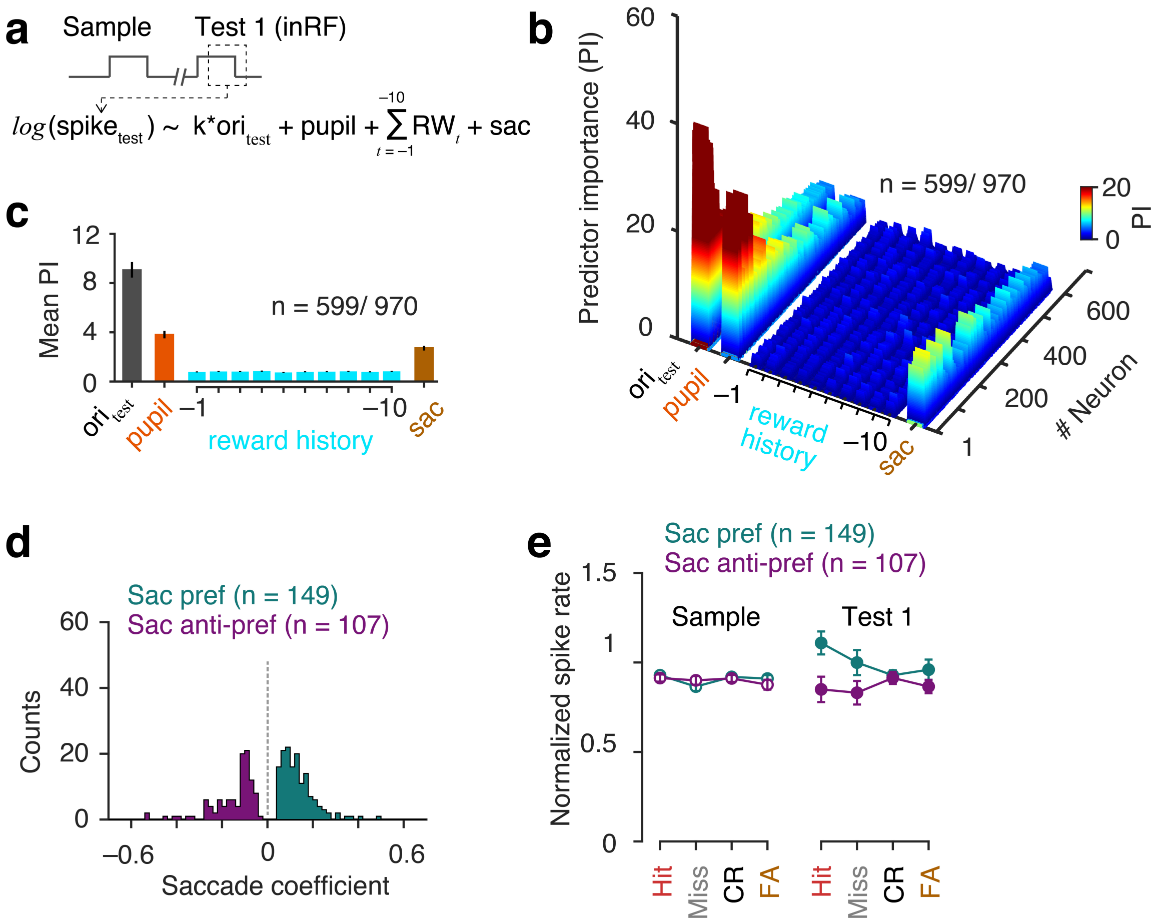


**Supplementary Figure 23. GLM fits of spike counts during test 1 period using complete model with saccade choice as a predictor variable. a** *Top*, Saccade GLM same as **Figure 6**. Spike counts were taken over 60-260 ms from sample onset. Predictor variables: product of test 1 stimulus orientation and neuron's orientation tuning filter, pupil area, reward history and saccade. Only a subset of trials was used for which test 1 stimulus appeared inside recorded neurons' RF and animals did not initiate any saccade before the 260 ms from test 1 on. **b, c** Predictor importance of individual neurons (b) and population average (significant fit, $p < 0.05$, n = 599/970; two-sided F test) (c). Data presented as mean with 95% confidence intervals. **d** Distribution of model fitted standardized coefficients of neurons with significant saccade coefficient ($p < 0.05$, n = 256; two-sided t-test). Neurons with positive saccade coefficient are referred as sac-preferred (n = 149) and with negative saccade coefficient are referred as sac anti-preferred (n = 107). **e** Observed mean normalized V4 spike rates of saccade selective neurons (sac preferred and sac anti-preferred) for different behavior choice trials during sample and test 1 stimulus presentations (60-260 ms from stimulus onset). H: hit, M: miss, R: correct rejection, F: false alarm. Data presented as mean with 95% confidence intervals (bootstrap, $n = 10^4$).
