# Supplementary material for: Single trial neuronal activity dynamics of attentional intensity in monkey visual area V4
Source: Nat Commun. 2021 Mar 31;12:2003. doi: 10.1038/s41467-021-22281-2 (PMC8012644; doi:10.1038/s41467-021-22281-2)
Supplement: Supplementary file 3 — Reporting Summary [file 41467_2021_22281_MOESM3_ESM.pdf]

nature research

Corresponding author(s): Supriya Ghosh

Last updated by author(s): Feb 26, 2021

Reporting Summary

Nature Research wishes to improve the reproducibility of the work that we publish. This form provides structure for consistency and transparency in reporting. For further information on Nature Research policies, see our Editorial Policies and the Editorial Policy Checklist.

Statistics

For all statistical analyses, confirm that the following items are present in the figure legend, table legend, main text, or Methods section.

n/a	Confirmed	
☐	☒	The exact sample size (*n*) for each experimental group/condition, given as a discrete number and unit of measurement
☐	☒	A statement on whether measurements were taken from distinct samples or whether the same sample was measured repeatedly
☐	☒	The statistical test(s) used AND whether they are one- or two-sided<br>*Only common tests should be described solely by name; describe more complex techniques in the Methods section.*
☐	☒	A description of all covariates tested
☐	☒	A description of any assumptions or corrections, such as tests of normality and adjustment for multiple comparisons
☐	☒	A full description of the statistical parameters including central tendency (e.g. means) or other basic estimates (e.g. regression coefficient) AND variation (e.g. standard deviation) or associated estimates of uncertainty (e.g. confidence intervals)
☐	☒	For null hypothesis testing, the test statistic (e.g. *F*, *t*, *r*) with confidence intervals, effect sizes, degrees of freedom and *P* value noted<br>*Give P values as exact values whenever suitable.*
☒	☐	For Bayesian analysis, information on the choice of priors and Markov chain Monte Carlo settings
☒	☐	For hierarchical and complex designs, identification of the appropriate level for tests and full reporting of outcomes
☐	☒	Estimates of effect sizes (e.g. Cohen's *d*, Pearson's *r*), indicating how they were calculated

Our web collection on statistics for biologists contains articles on many of the points above.

Software and code

Policy information about availability of computer code

Data collection	Neurophysiologycal data was collected using Cerebus Central (Blackrock Microsystems). Behavioral data was collected using custom software LabLib which is available from https://github.com/MaunsellLab/Lablib-Public-05-July-2016.git.
Data analysis	Neuronal spike sorting was done using Offline Sorter V 4.0 (Plexon Inc.). All other data analysis was performed using custome written codes in Matlab.

For manuscripts utilizing custom algorithms or software that are central to the research but not yet described in published literature, software must be made available to editors and reviewers. We strongly encourage code deposition in a community repository (e.g. GitHub). See the Nature Research guidelines for submitting code & software for further information.

Data

Policy information about availability of data

All manuscripts must include a data availability statement. This statement should provide the following information, where applicable:

- Accession codes, unique identifiers, or web links for publicly available datasets
- A list of figures that have associated raw data
- A description of any restrictions on data availability

Experimental data used in this study are available from the corresponding author upon request.

Field-specific reporting

Please select the one below that is the best fit for your research. If you are not sure, read the appropriate sections before making your selection.

- [x] Life sciences
- [ ] Behavioural & social sciences
- [ ] Ecological, evolutionary & environmental sciences

For a reference copy of the document with all sections, see nature.com/documents/nr-reporting-summary-flat.pdf

Life sciences study design

All studies must disclose on these points even when the disclosure is negative.

Sample size	Data are reported from a total of 970 units recorded from from 24 behavioral sessions in both monkeys (563 units, 15 sessions in monkey S; 407 units, 9 sessions in monkey P). Data was collected from 96 recording sites/animal in area V4. Data collection was based on numbers of units typically required to reach significance in neurophysiology studies on attention modulation (Cohen, M. R. & Maunsell, J. H. Nat. Neurosci. 12, 1594-1600 (2009))
Data exclusions	No data were excluded.
Replication	Results were successfully replicated in two monkeys, which is standard in non-human primate models
Randomization	Two monkeys participated in all experimental conditions. Each day, experimental conditions were presented in alternate block of trials sequentially (Material and Methods). Starting block was chosen in random order across days. Due to the chronic recordings, it is possible that some units were resampled across days. Any such resampling would have rarely involved identical stimulus configurations, as we adjusted the orientations and locations of the stimuli each day for a randomly selected unit.
Blinding	N/A: each day, initial experimental conditions were presented in a random order (Online Methods) as selected by a computer. All analysis of neural data was performed by analysis code applied to all conditions

Reporting for specific materials, systems and methods

We require information from authors about some types of materials, experimental systems and methods used in many studies. Here, indicate whether each material, system or method listed is relevant to your study. If you are not sure if a list item applies to your research, read the appropriate section before selecting a response.

Materials & experimental systems

n/a	Involved in the study	
x		Antibodies
x		Eukaryotic cell lines
x		Palaeontology and archaeology
	x	Animals and other organisms
x		Human research participants
x		Clinical data
x		Dual use research of concern

Methods

n/a	Involved in the study	
x		ChIP-seq
x		Flow cytometry
x		MRI-based neuroimaging

Animals and other organisms

Policy information about studies involving animals; ARRIVE guidelines recommended for reporting animal research

Laboratory animals	Two adult male rhesus monkeys (Macaca mulatta, 13 and 9 kg body weight, 10 and 12 years old) were used in this study.
Wild animals	No wild animals were used in this study
Field-collected samples	No field collected samples were used in the study
Ethics oversight	All experimental procedures were approved by the Institutional Animal Care and Use Committee at the University of Chicago and were in compliance with US National Institutes of Health guidelines.

Note that full information on the approval of the study protocol must also be provided in the manuscript.
